# Case Report: Mature tertiary lymphoid structures in a metastatic urothelial carcinoma patient with an exceptional response to sequential immune checkpoint inhibitor and antibody-drug conjugate therapy

**DOI:** 10.3389/fonc.2026.1907498

**Published:** 2026-09-03

**Authors:** Kanta Hori, Takuto Ogasawara, Yuka Mizue, Hiroko Asanuma, Naoki Shijubou, Akihiro Yamashita, Ryo Kato, Jun Furumido, Takashige Abe, Keisuke Sato, Yoshihiko Hirohashi

**Affiliations:** 1 Department of Urology, Asahikawa Kosei General Hospital, Hokkaido, Japan; 2 Departments of Pathology, Sapporo Medical University School of Medicine, Hokkaido, Japan; 3 Department of Renal and Genitourinary Surgery, Hokkaido University Graduate School of Medicine, Hokkaido, Japan; 4 Department of Pathology, Asahikawa Kosei General Hospital, Hokkaido, Japan

**Keywords:** antibody–drug conjugate, biomarker, immune checkpoint inhibitor, tertiary lymphoid structures, urothelial carcinoma

## Abstract

**Background:**

Biomarkers that predict durable benefit from immune checkpoint inhibitors (ICIs) and antibody-drug conjugates (ADCs) in metastatic urothelial carcinoma (mUC) remain limited. Tertiary lymphoid structures (TLS), particularly mature TLS (mTLS) characterized by follicular dendritic cell networks and germinal centers, may reflect a highly compartmentalized antitumor immune niche.

**Case presentation:**

A 77-year-old man with bladder cancer and lung metastases experienced disease progression after two cycles of gemcitabine/cisplatin. Pembrolizumab was initiated but discontinued after a single dose due to destructive thyroiditis; nevertheless, his lung metastases subsequently regressed. Because residual urothelial disease persisted in the bladder, enfortumab vedotin (EV) was initiated. A complete response (CR) was achieved after three cycles and maintained through 10 cycles, at which point treatment was stopped due to fatigue. The patient has remained recurrence-free for over 4 years since the initiation of pembrolizumab.

**Methods and results:**

Immunohistochemistry of the pre-treatment transurethral resection of bladder tumor (TUR-Bt) specimen (CD3, CD4, CD8, CD20, CD21, BCL6) revealed numerous TLS adjacent to the tumor, many of which met the criteria for mTLS (CD21+ FDC networks and BCL6+ germinal center B cells). A HALO-based digital pathology spatial analysis demonstrated a highly enriched B- and T-cell microenvironment, with the vast majority of these lymphocytes tightly compartmentalized within the mTLS regions. In an exploratory cohort of six additional sequentially treated mUC cases evaluated using the same methodology, mTLS were not detected, even among cases exhibiting a generalized T-cell infiltration.

**Conclusion:**

Pre-existing mTLS identified in routine TUR specimens reflect a highly compartmentalized immune niche—distinct from a simple “hot tumor” phenotype—that may help predict which patients are likely to achieve a durable benefit from sequential ICI and ADC therapy, potentially informing treatment selection and sequencing in mUC.

## Introduction

Metastatic urothelial carcinoma (mUC) remains a lethal disease despite major advances in systemic therapy. Immune checkpoint inhibitors (ICIs) and antibody-drug conjugates (ADCs), administered as monotherapies or in combination/sequence, are now widely used across multiple lines of mUC treatment. These agents have significantly improved outcomes for a subset of patients, including a minority who achieve deep and durable responses (1–4). Notably, pembrolizumab demonstrated an overall survival benefit versus chemotherapy in platinum-refractory advanced urothelial carcinoma (1), while enfortumab vedotin improved survival compared with chemotherapy in previously treated patients (2). More recently, the combination of enfortumab vedotin and pembrolizumab has emerged as a new first-line standard of care based on phase III evidence (3, 4). Nonetheless, in routine clinical practice, robust and broadly applicable biomarkers to reliably identify patients likely to derive meaningful benefit from ICIs and guide treatment sequencing remain scarce.

Tertiary lymphoid structures (TLS) are ectopic, lymph node-like aggregates that form in non-lymphoid tissues under conditions of chronic inflammation, including within or adjacent to tumors. TLS typically consist of organized T-cell zones and B-cell follicles, and their presence is associated with favorable prognosis and responsiveness to immunotherapy across several cancer types (5–8). Recent comprehensive reviews emphasize that the maturation status of TLS—ranging from immature aggregates to germinal center-containing structures—significantly influences their functional relevance and clinical implications (5–7). Of particular interest are mature TLS (mTLS), which exhibit features of lymphoid organogenesis such as follicular dendritic cell (FDC) networks and germinal center (GC) activity (5–7). These mature immune niches are thought to support local antigen presentation, clonal expansion, B-T cell cooperation, and the generation of tumor-reactive humoral and cellular immunity, thereby potentiating robust antitumor responses.

Here, we report the case of a patient with metastatic bladder cancer who achieved an exceptional, long-lasting complete response to sequential immunotherapy, consisting of a single dose of pembrolizumab followed by enfortumab vedotin. This remission has been sustained for more than four years since pembrolizumab initiation. Immunopathological analyses of the pre-treatment transurethral resection of bladder tumor (TUR-Bt) specimen revealed abundant TLS, most of which met the criteria for mTLS, presenting CD21-positive FDC networks and BCL6-positive germinal center B cells (5–7). Furthermore, an exploratory comparison using TUR specimens from additional mUC cases treated with similar sequential therapies at our institution highlighted the rarity of this phenotype. Our findings support the hypothesis that pre-existing mTLS in routine TUR material may mark an immune microenvironment highly permissive to durable immunotherapy benefits, thereby contributing to practical, pathology-based patient stratification in mUC.

## Case presentation

### Clinical history and diagnosis

**Figure 1:**
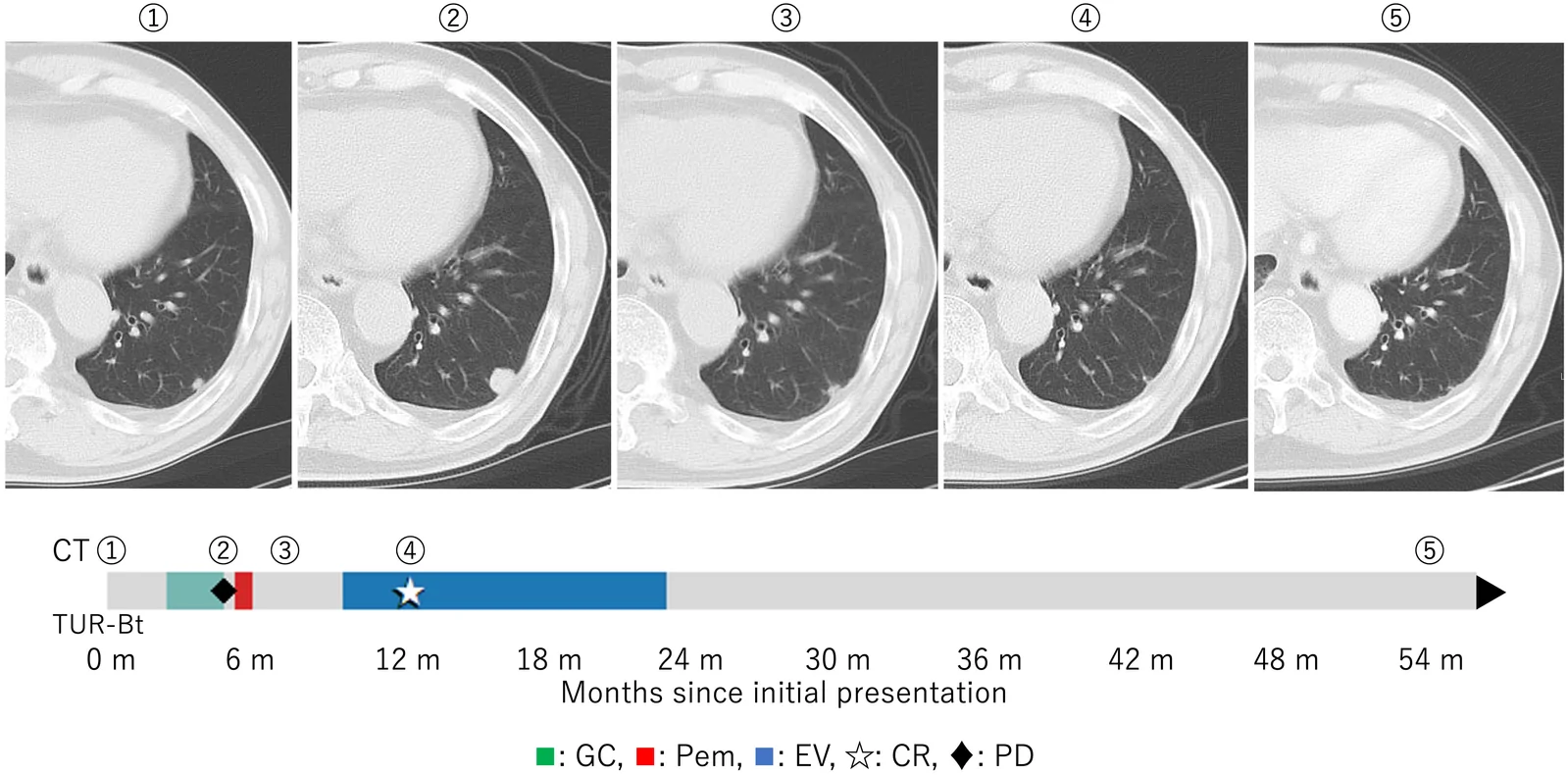
Clinical course and radiographic response. Timeline of treatment and computed tomography (CT) findings for pulmonary metastases from initial presentation to ongoing complete response (CR). Baseline CT showed a bladder tumor with lung metastases (①). Following transurethral resection of bladder tumor (TUR-Bt), pathology confirmed invasive high-grade urothelial carcinoma (≥pT2). The patient received gemcitabine plus cisplatin (GC) for two cycles and was assessed as progressive disease (PD) due to enlargement of lung metastases (②). Pembrolizumab (Pem) was administered as second-line therapy but discontinued after one dose because of destructive thyroiditis. Post-Pem CT demonstrated shrinkage of lung metastases (③). Enfortumab vedotin (EV) was subsequently initiated, and CR was achieved after three cycles (④); EV was continued for a total of 10 cycles with maintained CR. EV was discontinued due to adverse events, and the patient has since been followed under surveillance only. At >4.0 years from Pem initiation, CR remains ongoing (⑤). Symbols: squares indicate systemic therapies (GC, pembrolizumab, EV), diamond indicates PD, and star indicates CR.

A 77-year-old man presented with gross hematuria. Contrast-enhanced computed tomography (CT) revealed a primary bladder mass alongside multiple pulmonary lesions consistent with metastatic disease (**Figure 1**). The patient underwent a transurethral resection of the bladder tumor (TUR-Bt). Histopathological evaluation of the TUR specimen confirmed invasive, high-grade urothelial carcinoma that was at least muscle-invasive (≥pT2). Given the concomitant lung metastases, a definitive diagnosis of metastatic urothelial carcinoma was established.

### Treatment course and outcome

The patient received two cycles of gemcitabine plus cisplatin (GC) as first-line systemic therapy. However, restaging CT demonstrated the enlargement of the pulmonary metastases, indicating progressive disease (PD) (**Figure 1**). Second-line therapy with the anti-PD-1 antibody pembrolizumab was subsequently initiated. During the first cycle, the patient developed destructive thyroiditis, prompting the permanent discontinuation of pembrolizumab. Despite this early cessation, a follow-up CT scan revealed a reduction in the size of the pulmonary metastases (**Figure 1**). Nevertheless, residual urothelial disease persisted in the bladder. Given the presence of residual disease and the clinical availability of ADC therapy, enfortumab vedotin (EV) was initiated. Following three cycles of EV, all detectable lesions resolved on imaging and clinical assessment, achieving a complete response (CR) (**Figure 1**). EV was continued for a total of 10 cycles, maintaining the CR, but was eventually discontinued due to treatment-related adverse events, primarily fatigue. Since then, the patient has been managed with active surveillance without any further systemic therapy. Notably, despite receiving only a single dose of pembrolizumab and a finite course of EV, the patient has remained in durable remission for over four years from the initiation of pembrolizumab (and 4.5 years from the initial presentation), with no evidence of disease recurrence (**Figure 1**).

## Pathological and immunological assessment

### Specimen and histopathology

**Figure 2:**
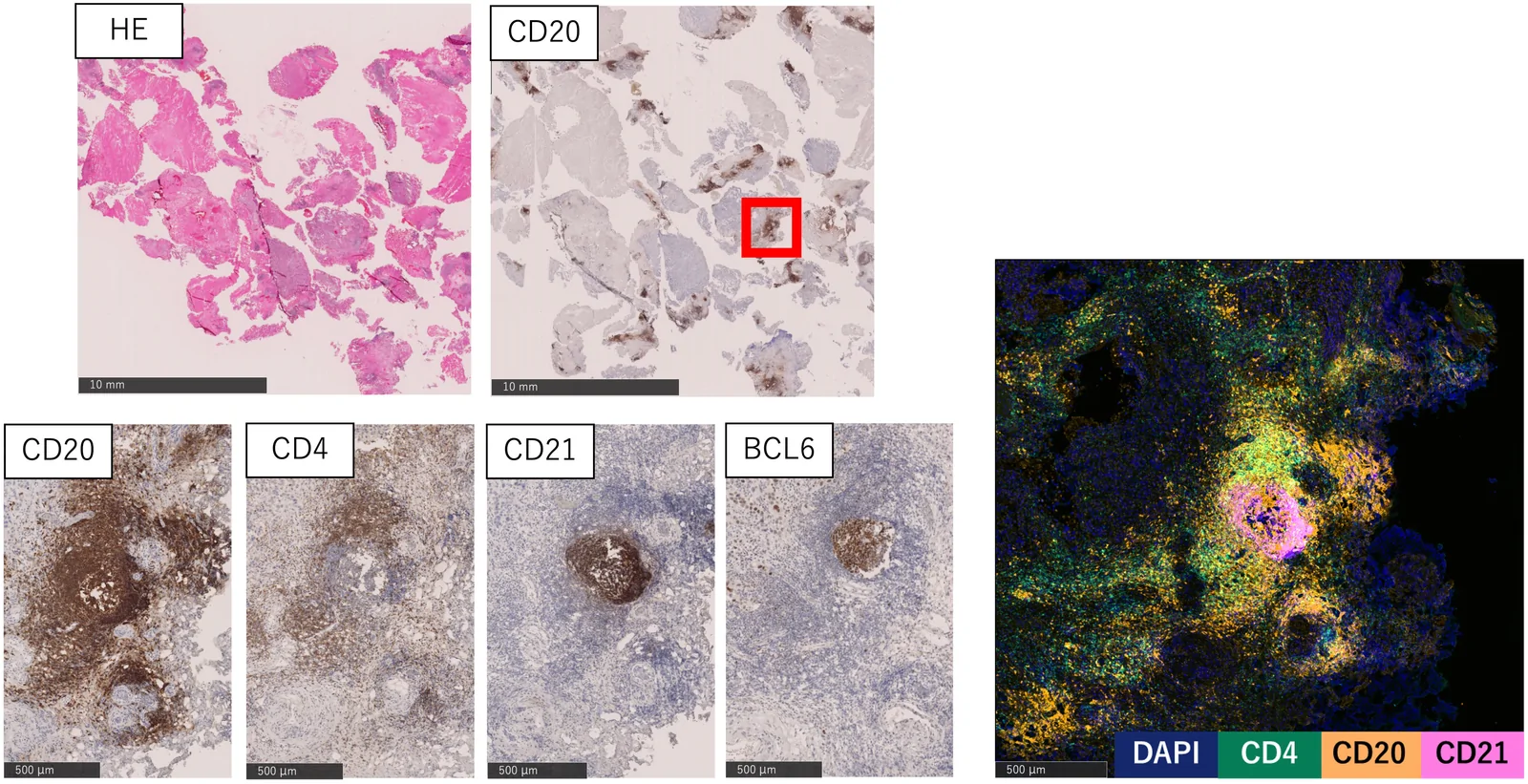
Mature tertiary lymphoid structures in the pre-treatment TUR-Bt specimen. Representative histopathological and immunohistochemical evaluation of the pre-treatment TUR-Bt specimen. Hematoxylin and eosin (H&E) staining shows prominent lymphoid aggregates adjacent to the tumor. CD20 immunostaining highlights B-cell–rich areas within these aggregates. Higher-magnification images of the highlighted region demonstrate organized immune architecture consistent with mature tertiary lymphoid structures (mTLS), supported by the presence of CD21-positive follicular dendritic cell networks and BCL6-positive germinal center B cells, together with CD4-positive T-cell components. Multiplex pseudo-fluorescence visualization generated by digital image analysis further depicts the spatial organization of these mTLS. Scale bars: 10 mm in the whole-slide H&E and CD20 images; 500 µm in all higher-magnification panels.

To explore the immunopathological correlates underlying this exceptional response, we retrospectively analyzed the pre-treatment TUR-Bt specimen after the institutional IRB approval (No. 2025001). Hematoxylin and eosin (H&E) sections confirmed invasive urothelial carcinoma. Beyond standard tumor morphology, our assessment focused on the immune architecture within the peritumoral and stromal regions, with specific attention to the presence and maturation status of TLS (**Figure 2**).

### Immunohistochemistry

Immunohistochemical staining was performed using a panel of markers: CD3 (pan-T cells), CD4 (helper T cells), CD8 (cytotoxic T cells), CD20 (B cells), CD21 (follicular dendritic cell networks), and BCL6 (germinal center B cells) (**Figure 2**). TLS were identified as discrete lymphoid aggregates exhibiting organized T- and B-cell zones. We operationally defined mature TLS (mTLS) as structures demonstrating both (i) CD21-positive FDC networks and (ii) BCL6-positive germinal center B cells within the lymphoid aggregate, indicative of germinal center-like maturation (5–7).

### Digital pathology analysis using HALO

To enable objective quantification and spatial profiling, we utilized the HALO image analysis platform (Indica Labs, Albuquerque, NM, USA). Multiplex pseudo-fluorescence images were generated from the immunohistochemistry slides using the Deconvolution Module, and the immune cell composition was quantified via the Classification Module. Such digital pathology approaches are increasingly utilized to standardize quantitative tissue biomarker assessments (9–11). Specifically, we calculated the proportion of the CD20-positive and CD3-positive areas within the tissue as an estimate of relative B-cell and T-cell abundance (expressed as %B cell and %T cell, derived from the CD20-positive and CD3-positive areas relative to the total analyzed tissue area). For spatial compartmentalization analysis, mTLS regions were manually annotated as discrete lymphoid aggregates exhibiting organized T- and B-cell zones under the guidance of a pathologist. To further evaluate lymphoid cell compartmentalization, we calculated the proportion of total tissue CD20-positive and CD3-positive cells localized specifically within these annotated mTLS regions (TLSB and TLST). In this index case, digital pathology revealed a conspicuously B-cell- and T-cell-rich immune landscape in the tumor-adjacent stroma. Multiple TLS were observed, many of which fulfilled the criteria for mTLS, displaying clear CD21-positive FDC networks and BCL6-positive germinal center areas (**Figure 2**).

### Key immunopathological findings in the index case

**Figure 3:**
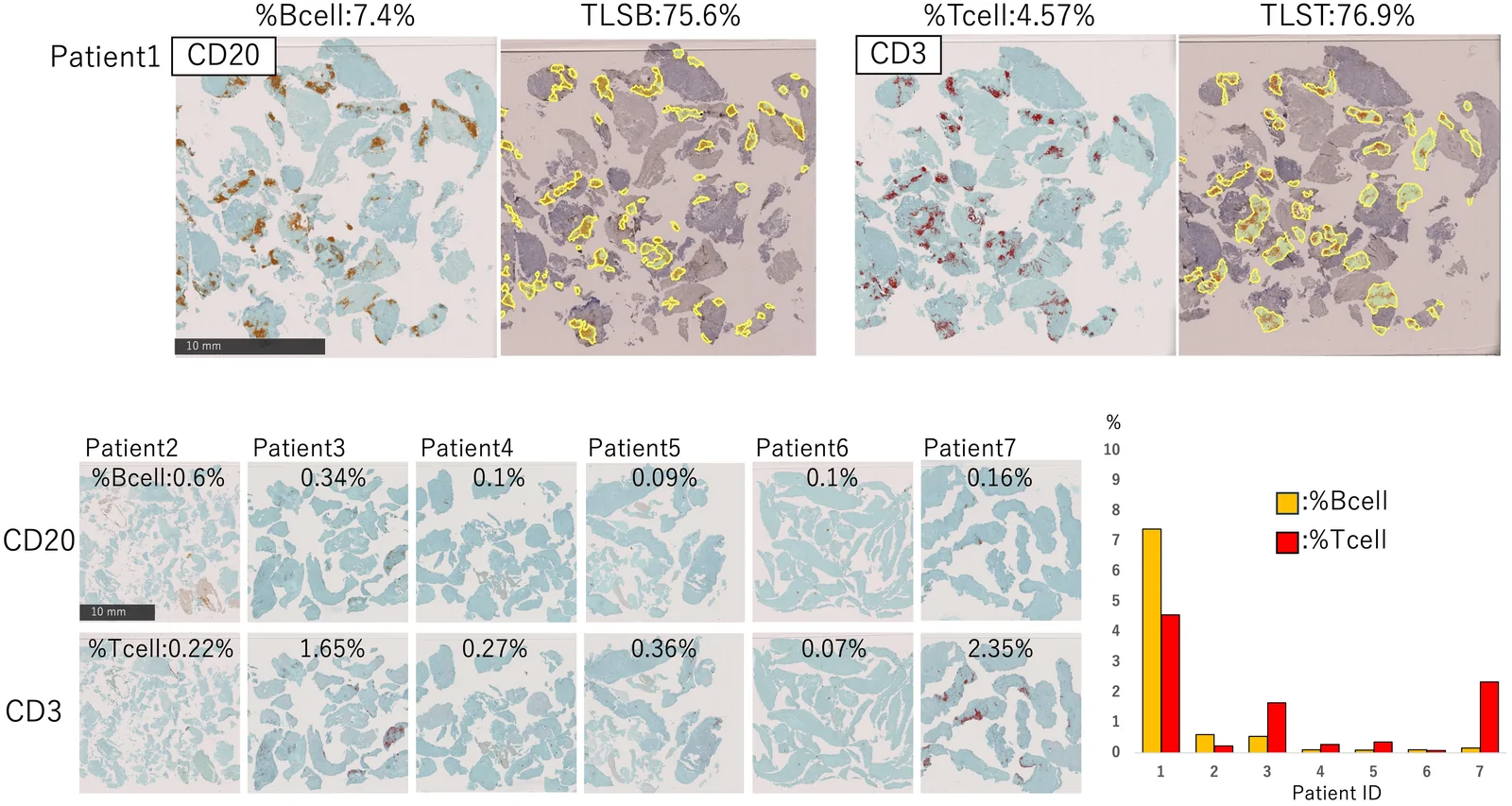
Quantitative and spatial correlative analysis of CD20+ B cells and CD3+ T cells across the seven-patient cohort. Representative whole-tissue digital layouts of CD20 (orange/brown) and CD3 (red) immunohistochemistry with corresponding HALO-derived overall tissue area fractions (%B cell and %T cell) for the seven-patient cohort. Patient ID 1 (index case) displays a markedly higher %B cell fraction (7.40%) alongside robust T-cell infiltration (4.57%) compared with the other cases. The lower panels illustrate the spatial correlative mapping analysis performed for Patient ID 1 to evaluate lymphoid cell compartmentalization. The mature tertiary lymphoid structure (mTLS) regions are annotated with yellow outlines. Compartmentalized cell recruitment is quantified and expressed as TLSB (the proportion of total tissue CD20+ B cells localized inside the mTLS regions; 75.6%) and TLST (the proportion of total tissue CD3+ T cells localized inside the mTLS regions; 76.9%), confirming a highly organized, mTLS-driven immune microenvironment rather than a generalized, non-specific inflamed phenotype. Scale bars: 10 mm.

The pre-treatment TUR specimen exhibited: (i) numerous TLS in the tumor-adjacent stromal compartment, composed of CD4-positive T cells and CD20-positive B cells with apparent zonal organization; (ii) a high frequency of mTLS, evidenced by CD21-positive FDC networks and BCL6-positive germinal center-like B-cell clusters (**Figure 2**); and (iii) a markedly enriched immune composition with overwhelmingly high %B cell and %T cell fractions based on HALO quantification (**Figure 3**). Crucially, our spatial correlative mapping revealed that the vast majority (approximately 75%) of both B cells and T cells were sequestered within these mTLS regions (TLSB: 75.6%; TLSB: 76.9%). Collectively, these findings indicate that the pre-existing immune microenvironment was not merely a generalized, non-specific inflamed phenotype (a simple “hot tumor”), but rather a highly compartmentalized, mTLS-driven immune niche capable of supporting antitumor immunity prior to systemic intervention.

### Exploratory institutional comparison cohort

**Figure 4:**
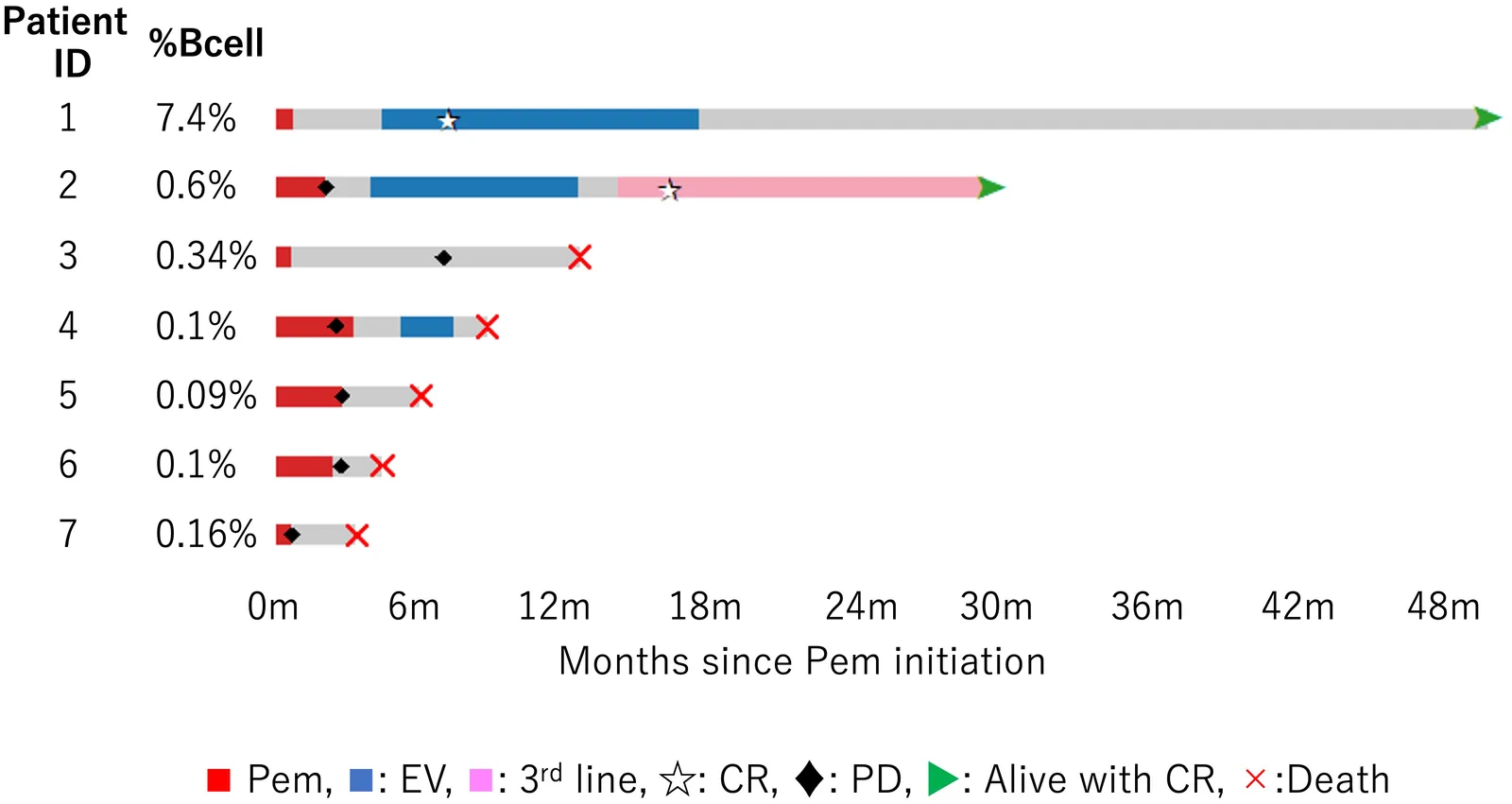
Swimmer plot of patients with metastatic urothelial carcinoma treated with sequential immunotherapy. Swimmer plot summarizing clinical courses of seven patients with metastatic urothelial carcinoma who received sequential immunotherapy. Time is plotted as months since pembrolizumab initiation. Bars indicate treatment exposure: pembrolizumab (red), enfortumab vedotin (blue), and third-line therapy (pink). Symbols denote clinical status: star, complete response (CR); diamond, progressive disease (PD); arrowhead, alive with CR; cross, death. Patient ID 1 (index case) demonstrated the most favorable outcome. For each patient, %B cell is shown as the proportion of CD20-positive area within the analyzed TUR specimen quantified by HALO-based digital pathology.

To contextualize the rarity of these findings and explore their potential clinical correlation, we evaluated pre-treatment TUR specimens from additional mUC patients who were treated with sequential immunotherapy at our institution. Out of 11 patients initially screened (defined as patients with metastatic bladder cancer treated with pembrolizumab between 01 December 2019 and 01 April 2026 at our institution), four cases were excluded due to insufficient quality of the pre-treatment TUR-Bt specimen for immunohistochemical evaluation. Consequently, seven eligible cases (including the index case) were available for analysis. In this exploratory cohort (n=6), the index case exhibited a substantially higher relative B-cell and T-cell abundance compared with the other cases (**Figure 3**). Interestingly, while some non-index cases (e.g., Patient ID 3 and 7) exhibited moderately high T-cell infiltration suggestive of a classical “hot tumor” phenotype, they lacked a correspondingly high B-cell fraction. Furthermore, area-evaluable mTLS were exclusively identified in the index case; they were completely absent in the other evaluated cases using the same methodology. The clinical outcomes for the cohort, depicted as a swimmer plot anchored to pembrolizumab initiation, are presented in **Figure 4**. The index case (ID 1) demonstrated the most favorable clinical course. While the small sample size and non-randomized nature of this comparison dictate that these observations be interpreted as hypothesis-generating, our findings suggest that highly compartmentalized mTLS, particularly with their robust B-cell clusters, create a specific microenvironment that drives durable responses to sequential immunotherapy. This compartmentalized structure may serve as a stronger prognostic marker in mUC than a simple ‘hot tumor’ phenotype.

## Discussion

Accumulating evidence across multiple solid tumors indicates that TLS—particularly mature, germinal center-containing TLS—are associated with a favorable prognosis and may correlate with benefit from immunotherapy (5–8, 12). TLS act as organized immunological hubs that facilitate local antigen presentation, the priming and expansion of tumor-reactive T cells, B-cell maturation, and the production of antitumor antibodies (5–8). The presence of FDC networks and BCL6-positive germinal center structures within mTLS implies an active, coordinated adaptive immune response within the tumor microenvironment. In urothelial carcinoma, several studies have linked TLS positivity to improved clinical outcomes, including in the context of immune checkpoint blockade (13, 14). Reports indicate that TLS can be found in a subset of muscle-invasive bladder cancers and correlate with survival and immune activation signatures (13, 15, 16). While previous research has suggested an association between TLS-related features and pembrolizumab outcomes in mUC (14), the specific frequency of *mature* TLS—and the feasibility of utilizing them as a routine pathology-based biomarker in standard TUR specimens—remains incompletely defined.

The present case is notable for three main reasons. First, the patient achieved an exceptional and durable CR following sequential immunotherapy, despite receiving only a single dose of pembrolizumab and a time-limited course of EV. Second, the pre-treatment TUR specimen contained abundant TLS, importantly featuring multiple mTLS with CD21-positive FDC networks and BCL6-positive germinal center B cells. Furthermore, spatial mapping confirmed that approximately 75% of both B and T cells were compartmentalized within these mTLS regions, distinguishing this highly compartmentalized architecture from a diffusely infiltrated “hot tumor”. Third, in our exploratory institutional comparison, mTLS were not detected in the TUR specimens of the other sequentially treated patients. Even though some patients exhibited moderately high T-cell infiltration (a simple “hot tumor”), their clinical outcomes were significantly inferior to the index case. This supports the notion that such highly compartmentalized immune architecture and the specific presence of TLS-associated B cells is uncommon in routine mUC TUR material and may be uniquely enriched among exceptional responders.

Clinically, this durable response is striking compared to typical outcomes for later-line systemic therapies in mUC. While pembrolizumab and EV have both demonstrated survival benefits in their respective settings (1, 2), long-term complete responses remain relatively rare. In our case, despite the early discontinuation of pembrolizumab due to destructive thyroiditis, pulmonary metastases shrank, suggesting that even brief ICI exposure triggered a meaningful antitumor immune cascade. The subsequent administration of EV likely facilitated the complete eradication of residual disease, acting synergistically with an already primed immune microenvironment. Importantly, because the patient received only a single dose of pembrolizumab before switching to EV, we cannot definitively conclude from this case whether the mature TLS marked responsiveness to the checkpoint inhibitor, the ADC, or the specific sequence itself. Rather than proposing mTLS as a demonstrated ADC biomarker, we present the hypothesis that mTLS represents an ICI-primed immune competence that potentially synergizes with subsequent therapies. Preclinical data suggests that ADCs exert immunomodulatory effects, including the induction of immunogenic cell death and the enhancement of immune infiltration, providing a strong biological rationale for synergy with immune checkpoint blockade (17–19). Although causality cannot be definitively proven from a single case, the clinical timeline aligns with a model wherein pre-existing immune competence—reflected by abundant mTLS—enabled rapid, durable immune-mediated tumor control, with ADC therapy delivering supplementary cytoreduction and immune reinforcement.

Mechanistically, mTLS may provide a localized sanctuary for sustained immune surveillance. Germinal center reactions supported by FDC networks generate affinity-matured B cells and facilitate critical B-T cell crosstalk (5–7). Antibody responses may augment antigen presentation and Fc receptor-mediated tumor killing, while the broader TLS ecosystem sustains effector and memory T-cell responses (5–8). In the context of ICI therapy, this structured immune niche could amplify the reinvigoration of exhausted T cells. The presence of these processes prior to treatment suggests that routine diagnostic tissue evaluation could identify tumors that are already “immunologically organized” and primed to respond.

Our exploratory comparison also underscores a practical advantage: TUR-Bt specimens are routinely obtained during standard bladder cancer care, making them an accessible resource for assessing TLS as a prognostic marker (20). If mTLS and related immune features can be robustly evaluated using standardized immunohistochemical panels and digital pathology workflows, routine pathology-based stratification could become highly feasible (9–11). In an era of rapidly expanding ICI and ADC combinations (3, 4, 21), such stratification could be invaluable for informing treatment selection and sequencing.

This report has several important limitations. First, we evaluated TLS/mTLS using pre-treatment TUR-Bt specimens from the primary bladder tumor, whereas the major therapeutic responses were assessed in metastatic lesions (lung metastases). Therefore, extrapolating immune architecture in the primary tumor to estimate drug sensitivity and response durability in metastatic sites may be limited by inter-site heterogeneity; as tissue-level immune architecture might not necessarily travel well between primary and metastatic sites, the microenvironment of the primary tumor may not fully reflect that of the distant metastases. As a single-case observation supplemented by a small exploratory cohort, our findings are subject to selection bias and confounding clinical variables. Because the patient received sequential therapies, the sustained response cannot be attributed exclusively to either pembrolizumab or EV. Furthermore, tissue-based TLS assessment faces inherent methodological challenges, including spatial heterogeneity and the limited sampling scope of TUR specimens (5–7). Finally, our quantification metric (%B cell by CD20-positive area) serves as a pragmatic proxy but does not capture detailed B-cell phenotypic states, clonality, or functional specificity. Despite these limitations, the striking convergence of an exceptional clinical trajectory with profound pre-treatment immune organization strongly supports the hypothesis that mTLS in routine TUR material is a promising candidate biomarker for exceptional immunotherapy responses in mUC. Larger, prospective studies utilizing standardized digital pathology pipelines and clear clinical endpoints are warranted to validate the clinical utility of mTLS as a candidate biomarker in urothelial carcinoma.

## Data Availability

The original contributions presented in the study are included in the article/supplementary material. Further inquiries can be directed to the corresponding author.

## References

[1] Bellmunt J de Wit R Vaughn DJ Fradet Y Lee JL Fong L . Pembrolizumab as second-line therapy for advanced urothelial carcinoma. N Engl J Med. (2017) 376:1015–26. doi: 10.1056/NEJMoa1613683 28212060 PMC5635424

[2] Powles T Rosenberg JE Sonpavde GP Loriot Y Durán I Lee JL . Enfortumab vedotin in previously treated advanced urothelial carcinoma. N Engl J Med. (2021) 384:1125–35. doi: 10.1056/NEJMoa2035807 33577729 PMC8450892

[3] Powles T Valderrama BP Gupta S Bedke J Kikuchi E Hoffman-Censits J . Enfortumab vedotin and pembrolizumab in untreated advanced urothelial cancer. N Engl J Med. (2024) 390:875–88. doi: 10.1056/NEJMoa2312117 38446675

[4] Powles T Bellmunt J Comperat E De Santis M Huddart R Loriot Y . ESMO clinical practice guideline interim update on first-line therapy in advanced urothelial carcinoma. Ann Oncol. (2024) 35:485–90. doi: 10.1016/j.annonc.2024.03.001 38490358

[5] Sautès-Fridman C Petitprez F Calderaro J Fridman WH . Tertiary lymphoid structures in the era of cancer immunotherapy. Nat Rev Cancer. (2019) 19:307–25. doi: 10.1038/s41568-019-0144-6 31092904

[6] Teillaud JL Houel A Panouillot M Riffard C Dieu-Nosjean MC . Tertiary lymphoid structures in anticancer immunity. Nat Rev Cancer. (2024) 24:629–46. doi: 10.1038/s41568-024-00728-0 39117919

[7] Fridman WH Meylan M Pupier G Calvez A Hernandez I Sautès-Fridman C . Tertiary lymphoid structures and B cells: An intratumoral immunity cycle. Immunity. (2023) 56:2254–69. doi: 10.1016/j.immuni.2023.08.009 37699391

[8] Li L Guo Y Gong B Wang S Wang MM Yang L . Association between tertiary lymphoid structures and clinical outcomes in cancer patients treated with immune checkpoint inhibitors: an updated meta-analysis. Front Immunol. (2024) 15:1385802. doi: 10.3389/fimmu.2024.1385802 38994363 PMC11236553

[9] Lara H Li Z Abels E Aeffner F Bui MM ElGabry EA . Quantitative image analysis for tissue biomarker use: A white paper from the Digital Pathology Association. Appl Immunohistochem Mol Morphol. (2021) 29:479–93. doi: 10.1097/PAI.0000000000000930 33734106 PMC8354563

[10] Taube JM Sunshine JC Angelo M Akturk G Eminizer M Engle LL . Society for Immunotherapy of Cancer: updates and best practices for multiplex immunohistochemistry (IHC) and immunofluorescence (IF) image analysis and data sharing. J Immunother Cancer. (2025) 13:e008875. doi: 10.1136/jitc-2024-008875 39779210 PMC11749220

[11] Viratham Pulsawatdi A Craig SG Bingham V McCombe K Humphries MP Senevirathne S . A robust multiplex immunofluorescence and digital pathology workflow for the characterisation of the tumour immune microenvironment. Mol Oncol. (2020) 14:2384–402. doi: 10.1002/1878-0261.12764 32671911 PMC7530793

[12] Jiang B Wu Z Zhang Y Yang X . Associations between tertiary lymphoid structure density and immune checkpoint inhibitor efficacy in solid tumors: systematic review and meta-analysis. Front Immunol. (2024) 15:1414884. doi: 10.3389/fimmu.2024.1414884 39544934 PMC11560435

[13] Zhou L Xu B Liu Y Wang Z . Tertiary lymphoid structure signatures are associated with survival and immunotherapy response in muscle-invasive bladder cancer. OncoImmunology. (2021) 10:1915574. doi: 10.1080/2162402x.2021.1915574 34104539 PMC8143239

[14] Komura K Tokushige S Ishida M Hirosuna K Yamazaki S Nishimura K . Tertiary lymphoid structure and neutrophil-lymphocyte ratio coordinately predict outcome of pembrolizumab. Cancer Sci. (2023) 114:4622–31. doi: 10.1111/cas.15976 37752769 PMC10728008

[15] Teng X Chen Z Bai Y Cao H Zhang J Xu L . Tertiary lymphoid structures as independent predictors of favorable prognosis in muscle-invasive bladder cancer. Cancer Med. (2025) 14:e70978. doi: 10.1002/cam4.70978 40396416 PMC12093152

[16] Lin J Jiang S Chen B Du Y Qin C Song Y . Tertiary lymphoid structures are linked to enhanced antitumor immunity and better prognosis in muscle-invasive bladder cancer. Adv Sci (Weinh). (2025) 12:e2410998. doi: 10.1002/advs.202410998 39739621 PMC11831474

[17] Nicolò E Giugliano F Ascione L Tarantino P Corti C Tolaney SM . Combining antibody-drug conjugates with immune checkpoint inhibitors in solid tumors: rationale and clinical progress. Ann Oncol. (2022) 33:472–84. doi: 10.1016/j.annonc.2021.12.014 35026411 PMC9338780

[18] Fu Z Li S Han S Shi C Zhang Y . Antibody drug conjugate: the "biological missile" for targeted cancer therapy. Signal Transduct Target Ther. (2022) 7:93. doi: 10.1038/s41392-022-00947-7 35318309 PMC8941077

[19] Yajima S Masuda H . Immune checkpoint inhibitors and antibody-drug conjugates in urothelial carcinoma: current landscape and future directions. Cancers (Basel). (2025) 17:1594. doi: 10.3390/cancers17091594 40361519 PMC12071276

[20] Yilmaz F Sagir S . Prognostic and predictive value of tertiary lymphoid structures in TURBT materials: Should it be seated in the routine pathological examination, and can it be used in deciding on the treatment method? Urol Oncol. (2024) 42:450.e13–450.e22. doi: 10.1016/j.urolonc.2024.06.010 39089974

[21] Powles T Park SH Voog E Caserta C Valderrama BP Gurney H . Avelumab maintenance therapy for advanced or metastatic urothelial carcinoma. N Engl J Med. (2020) 383:1218–30. doi: 10.1056/NEJMoa2002788 32945632

